# The Turnover Intention among Grassroots Family Planning Staff in the Context of China’s Universal Two-Child Policy: A Case Study of the Xi’an City

**DOI:** 10.3390/ijerph17228478

**Published:** 2020-11-16

**Authors:** Ling Xu, Jianghua Liu

**Affiliations:** 1 The School of Public Policy & Administration, Xi’an Jiaotong University, Xi’an 710049, China; xl908522899@stu.xjtu.edu.cn; 2 The Institute for Population & Development Studies, Xi’an Jiaotong University, Xi’an 710049, China

**Keywords:** family planning, turnover intention, affective organizational commitment, overall job satisfaction, two-child policy, the theory of planned behavior, Maslow’s theory of human motivation

## Abstract

The introduction of the universal two-child policy in 2016 marked a major social transition in China and raised a requirement for family planning services; however, the turnover in family planning staff poses a challenge to satisfying the requirement. Thus far, after implementation of the policy, there have been few surveys investigating turnover intention and the underlying motivations in grassroots family planning staff, the major component of China’s family planning system. A survey conducted in Xi’an in 2019 shows that nearly one in three grassroots members had an explicit or implicit turnover intention. Basically consistent with our conceptual framework, the structural equation modeling further indicates that the affective organizational commitment had the largest direct effect on turnover intention and also partly mediated effects of other significant factors (ranked by the size of total effect): Age, specific job satisfactions (i.e., satisfactions with job prospects, relationship with colleagues, and working environment), frequency of working overtime, length of service, and opportunity of professional training. As predicted, turnover behavior in colleagues also directly affected turnover intention in such staff. The above findings have important policy implications for the sustainable development of family planning work in China.

## 1. Introduction

As an effective preventive health measure, family planning has played an important role in promoting the quality of life of mothers and children in China, the most populous developing country in the world [1,2]. After more than 30 years of the so-called one-child family planning policy, the Chinese central government made a resolution to implement a universal two-child policy, i.e., (almost) all married couples were allowed to have two children, at the end of 2015. In making the resolution, the Chinese central government required that there should be a transition in the family planning work from being administration-oriented to being service-oriented, e.g., services such as prenatal and postnatal cares and reproductive health education and consultation should be assured [2]. It seems that such a requirement was rather visionary; e.g., compared to the time before implementing the two-child policy, abortions have increased by more than 3 million and reached a size close to 10 million since 2016 [3]. However, there is a major challenge to satisfying the requirement. As the major component of China’s family planning system, the grassroots family planning staff directly contact reproductive-aged women, but the stability of their jobs is by no means optimistic [4]; e.g., one in six grassroots family planning workers explicitly had a turnover intention in the Beijing city [5]. In light of the challenge, it makes important sense to explore the grassroots family planning staff’s turnover intention and the factors influencing it.

Previous studies have shown that a series of factors such as age, education, income, work stresses, organizational culture, and external organizational prestige could have an influence on employees’ turnover intention [6,7,8,9,10]. Further, two major job attitudes, i.e., job satisfaction and organizational commitment, have been shown to be relevant to employees’ turnover intention and mediate the effects of the above-mentioned factors on turnover intention [6,10,11,12,13,14,15]. Such findings based on the practices in other sectors also get supported in those studies on health workers in China. For instance, there was a significant negative association between job satisfaction and turnover intention among rural doctors in China [16]. In Guangdong Province of China, work stress and work–family conflict significantly predicted higher turnover intention among physicians, and such effects were partly mediated by job satisfaction [17]. The implementation of the two-child policy has also brought challenges to the work of pediatric nurses in Mainland China, and a recent study showed that work stress influenced turnover intention through its effects on organizational commitment and job satisfaction [18].

In spite of such rich survey-based findings of turnover intention in other health workers (i.e., doctors, nurses, etc.), most of the previous studies on turnover in grassroots family planning workers in China have only qualitatively discussed the topic [4]. Among those few survey-based analyses, the focus was actually not directly on turnover, but on the salary of grassroots staff or operating expense for family planning work [19,20,21]. The situation changed somewhat in recent years, partly due to the necessity of satisfying the requirements of the universal two-child policy. Just before the implementation of the policy, a survey of grassroots family planning staff in Shandong, Hubei, and Gansu provinces indicated that almost half of them had an explicit or implicit turnover intention, which was significantly influenced by their salary, old-security insurance, and working challenges [4]. The above result was basically supported by another study based on a survey conducted after the implementation of the two-child policy: In Beijing, the main factors influencing turnover intention in grassroots family planning staff included professional training opportunity, income satisfaction, and perceived difficulties in work [5].

So far, we are still lacking empirical studies to systematically investigate motivations underlying turnover intention in grassroots family planning staff. Especially, we do not know how the well-known job attitudes including affective organizational commitment and job satisfaction will explain the turnover intention among them. To fill the gap, we conduct the current study to analyze the paths of how individual and organizational factors were associated with job attitudes and turnover intention in grassroots family planning staff. Based on analysis results, some measures to promote the sustainable development of family planning work in China will be discussed.

## 2. Materials and Methods

### 2.1. The Evolution of China’s Family Planning Policies in Recent Years

From the 1980s to 2013, China implemented a so-called one-child family planning policy to control population growth and improve population quality, and the governments encouraged late marriage and childbearing and advocated that a couple had only one child [22]. The policy was implemented rather strictly in urban areas, but couples in rural areas were allowed to have two or more children under some conditions—e.g., the first child was a girl or the couples were of minority ethnic groups—in most provinces. The policy displayed dual effects: On the one hand, it played an important role in promoting China’s economic development and reducing the pressure of population on resources and the environment; on the other hand, it also brought some undesirable problems for a balanced development of population such as very low fertility, labor-force shortage, and quick population ageing [19,23,24]. Thus, the Central Committee of the Communist Party of China (CPC) made a decision to implement a selective two-child family planning policy on 12 November 2013 [25]. The national population sample surveys indicated that the policy effect on births was not satisfactory at all: The births in 2015 were even less than those in 2014 by about 330 thousand or 2% [26]. Thus, the Chinese government decided to implement a universal two-child policy, i.e., almost all couples were allowed to have two children (note that some remarried couples may not be allowed to do so), on 29 October 2015 [27] and the implementation started simultaneously in all provinces from 1 January 2016.

### 2.2. The Grassroots Family Planning Staff in China

As the main force in implementing the national basic policy of family planning, the grassroots family planning staff members engage in family planning work in urban communities and rural villages (the lowest administrative unit) in China; generally, each community or village is equipped with at least one family planning worker, who is generally a woman. Before the implementation of the two-child policy, their daily works were administrative in nature: Propaganda of family planning laws and regulations, supervision of long-acting contraception among reproductive-aged women, helping local government to collect the so-called social compensation fee for penalizing extra births that were not allowed by the previous one-child policy, etc. However, with the coming of the two-child policy, family planning work was required to turn service-oriented: Encouragement to having two children, helping families with special difficulties like losing the single child, publicity of reproductive health knowledge, guiding the reproductive-aged couples to arrange childbirth in a responsible and planned manner, distribution of contraceptive drugs and condoms, coordinating family planning surgeries like insertion or removal of intrauterine devices (IUDs), provision of healthcare to women from prenatal to postnatal periods, family health promotion, counting population and its changes (mainly births and deaths), etc. [2,28,29,30]. It was also suggested that future services be extended to other family members—e.g., husband with infertility problems—rather than being restricted to women [28]. The above emphasis of services over administration was expressed vividly in official words: The two-child policy is of considerable importance to the well-being of hundreds of millions of families, and to implement the policy well, the family planning services should be strengthened and become richer in content [31]. Evidently, the network of millions of family planning staff makes up the human capital indispensable for carrying out such services [28,29].

Besides the above regular duties, they are sometimes assigned additional works such as coordinating free physical examinations for the elderly and chronic disease intervention. Despite the significant work that needs to be done, they receive a low salary, as they are not regular public servants (Table 1); rather, they are hired by local government as temporary contractors, although the contract can be extended after expiration (for the problem of low salary in other provinces like Gansu, Hebei, Hubei, Jilin, and Shandong, see refs. [4,30,32,33]). Thus, to a large extent, they take the job of family planning services, not because it brings high income, but because it brings other benefits, e.g., working place is close to family and such a post allows a combination of work and family.

### 2.3. The Conceptual Model and Hypothesis Construction

**Figure 1:**
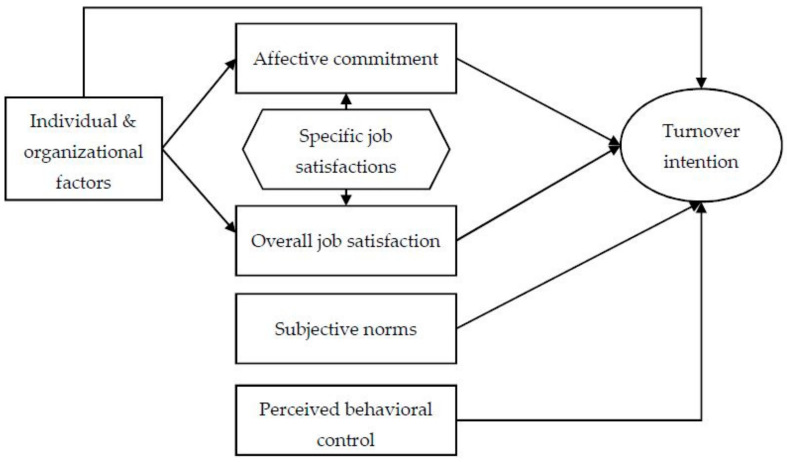
The conceptual framework of turnover intention.

Figure 1 shows the framework used in the current study. It was based on Ajzen’s theory of planned behavior [34], Maslow’s theory of human motivation [35], as well as a series of frameworks of organizational behavior. 

According to the theory of planned behavior, the direct antecedents of turnover intention will be job attitudes, subjective norms, and perceived behavioral control. Here, subjective norms can be either descriptive (actual behaviors of important others) or injunctive (opinions of important others toward the behavior under question). The perceived behavioral control refers to the ease or difficulty in performing a behavior; it is somewhat equivalent to continuance commitment in Allen and Meyer’s model [12]. Among these three predictors, the attitudes have received the most attention in studying turnover intention/behavior.

Strictly speaking, the attitudes that are directly relevant to turnover intention should be (explicitly) expressed attitudes toward turnover behavior (an example was given by ref. [36]). However, according to Maslow’s theory, the more fundamental motivations underlying leaving an organization stems from unmet needs, which can be either affective or cognitive ones. When such unmet needs arise, an intention to quit the current post and find an alternative one will naturally develop [8,13,37]. One of the best measurements of whether affective needs are met is affective organizational commitment, and the corresponding one for cognitive needs is job satisfaction; these two attitudes constitute the major precursors of the direct attitudes toward turnover behavior [36,38]. In practice, job satisfaction can be measured with a single item (e.g., overall job satisfaction) or a scale and is expected to be influenced by a series of specific satisfactions, e.g., satisfactions with income, working environment, and job prospects. Affective organizational commitment is generally measured with a scale, e.g., Allen and Meyer’s scale, which might include those items on being proud in the organization, esteem, and sense of self-actualization; evidently, such items correspond to higher-level needs in Maslow’s theory. Note that affective commitment is also expected to be influenced by the specific satisfactions above, as they are related to working experience, the major factor underlying affective commitment [39]. We now have the following set of hypotheses on job attitudes:

**Hypothesis 1a** (**H1a**). *Higher affective organizational commitment will be associated with a lower turnover intention in family planning staff*.

**Hypothesis 1b** (**H1b**). *Higher job satisfaction will also be associated with a lower turnover intention in family planning staff*.

**Hypothesis 1c** (**H1c**). *Both affective organizational commitment and job satisfaction will be influenced by specific satisfactions, e.g., satisfaction with salary, satisfaction with working environment, and satisfaction with work perspective*.

Second, both the theory of planned behavior and the above models of organizational behaviors suggest that affective commitment and job satisfaction will mediate (at least partly) the effects of various individual and organizational factors. However, some theoretical or empirical studies suggest that these factors can also have a direct impact on behavioral intention [6,9,40]. These factors could include previously well-studied ones like age, education, length of service and income, as well as those factors that were presumably typical in Chinese health workers like professional training and working overtime [4,5,39,41,42,43,44]. For example, increase in age was associated with a lower turnover intention in professional managers, and such a negative effect was only partly mediated by affective commitment [39]. Another study of professional managers in China showed that once affective commitment and job satisfaction were included in the model, the positive effect of education and the negative effect of income on turnover intention were much reduced, but education’s effect was still significant [13]. We now have the second set of hypotheses:

**Hypothesis 2a** (**H2a**). *The effects of individual and organizational factors on turnover intention will be partly mediated by affective organizational commitment and job satisfaction*.

**Hypothesis 2b** (**H2b**). *The individual and organizational factors also have some direct effects on turnover intention among family planning staff*.

According to the theory of planned behavior, we have the third set of hypotheses:

**Hypothesis 3a** (**H3a**). *The turnover intention will be stronger, either when the important others have an unsupportive opinion toward the current post or when the actual turnover increases in colleagues*.

**Hypothesis 3b** (**H3c**). *The difficulties that are encountered in work will be associated with higher turnover intention, but those factors that restrain quitting the current job will attenuate turnover intention in family planning staff*.

### 2.4. Data Collection

Our survey was conducted in the Xi’an metropolitan area, Shaanxi Province, a province in west China, from July to September 2019. Before the questionnaire survey, a two-stage sampling was conducted. First, three of 15 administrative districts—i.e., Beilin, Weiyang, and Yanliang—were selected based on judgment: They were believed to be able to represent three types of districts, i.e., main-city district, inner-suburb district, and outer-suburb district. Second, all grassroots family planning staff working in the three districts were included in the sample. In total, 330 staff were contacted and interviewed by telephone and 305 effective questionnaires were successfully collected. The authors promised all subjects that their answer would be kept fully confidential and they then gave their informed consent for inclusion before participating in the survey.

The survey was conducted in accordance with the Declaration of Helsinki and was approved by the Biomedical Ethics Committee of Xi’an Jiaotong University (No. 20202).

### 2.5. Measures

In the questionnaire design, we first conducted in-depth qualitative interviews with nine staff members to list important items that needed to be considered. We also borrowed some measurements used in the HBAT—i.e., HBAT Industries, a manufacturer of paper products in U.S.—organizational behavior study in designing items for measuring turnover intention, job satisfaction, and affective organizational commitment [45].

The questions (items) on turnover intention were as follows: Item 1, “You are actively looking for another job” and item 2, “You are interested in finding a new job this year or next year” (Options: 1—disagree; 2—somewhat disagree; 3—neither agree nor disagree; 4—relatively agree; 5—strongly agree).

The following sets of questions were asked to measure predictors of the turnover intention: (1) Individual characteristics at the time of survey: Place of residence, personal age, and education level. Note that as almost all members were women, we did not use personal gender as a predictor (Table 1). (2) Working-related measures: Length of service, monthly salary, frequency of working overtime, and opportunity of professional training. (3) Affective organizational commitment: “The engagement in health and family planning services brings me a sense of accomplishment”; “I am proud to tell others that I am engaging in health and family planning services”; “I feel respected in health and family planning services.” (4) Overall job satisfaction: “Overall, how satisfied are you with your current job?” (5) Specific job satisfactions (with income, working environment, colleague relationship and job prospects), e.g., “You are satisfied with the prospects of family planning work.” (6) Subjective norm: “In recent three years, how often have you seen other family planning staff quitting their posts in your working street?” (7) Factors limiting or promoting quitting the current job: “You take your current job, because the working place is close to your family”; “You take your current job, because it allows a combination of work and care of your children”; “You have encountered the following difficulties in your work” (six in total). Appendix A Table A1 gives more details about the coding of these predictors.

Table 1 shows descriptive statistics of all the variables mentioned above.

### 2.6. Data Analysis

Based on the hypotheses raised, we tested whether individual and organizational factors directly and indirectly—via affective commitment and overall job satisfaction—affected turnover intention, and whether norm and constraint factors directly affected turnover intention in grassroots family planning staff. Here, both affective commitment and turnover intention were latent variables that were not directly measured but indirectly measured through a series of directly observable indicators. Evidently, the structural equation model (SEM) was the suitable tool to conduct the analysis. The computation platform was R and the statistical package used was lavaan, which adopts a maximum likelihood estimation [46,47] (note: *lavaan* used a special scaling of variables in the factor analysis of latent variables, i.e., “the factor loading of the first indicator of a latent variable is fixed to 1”). We used two incremental fit indices (i.e., Comparative Fit Index or CFI and Tucker–Lewis Index or TLI; both indices measure how well the estimated model fits relative to the alternative null model assuming that all observed variables are uncorrelated) and one absolute fit index (i.e., root-mean-square error of approximation, RMSEA) to assess the goodness-of-fit of the SEM model. The result shows that CFI = 0.949 > 0.9, RMSEA = 0.047 < 0.08, TLI = 0.922 > 0.9, and thus, our model fitted well the sample (for the cut-off criteria of such indices, see refs. [45,48]).

As the survey was conducted in a self-reported manner, Harman’s one-factor analysis was used to test whether there was common method bias with our measurements: The confirmatory factor analysis of all subjective items indicated that the first factor explained about 25% of total variance in these items; the additional factor analysis of items for two latent constructs (i.e., turnover intention and affective commitment) indicated that the two-factor model ($\chi^{2}$ = 1.06, *p* < 0.3; TLI = 0.999, RMSEA = 0.014) was much better than the one-factor model ($\chi^{2}$ = 143.34, *p* < 3.5 × 10^−29^; TLI = 0.453, RMSEA = 0.301). Thus, there was some common method bias, but it was not serious [49,50].

## 3. Results

### 3.1. Descriptive Statistics

We first report the descriptive statistics of the variables included in SEM analysis (Table 1). As far as it was concerned with item 1 of turnover intention, the mean was 2.00, indicating that most of the grassroots family planning staff were not actively searching for a new job. However, the turnover intention was by no means negligible: 11.8% of staff selected the options of “relatively agree” or “strongly agree”; if we considered the option of “neither agree nor disagree,” the cumulative proportion arrived at 37%.

Only five among 305 members were men; all others were women. Around 68% of staff members came from an urban area (main-city or inner suburb areas) and 32% of staff members came from a rural area; the proportions here were basically consistent with the proportion of urbanization—around 72%—in the Xi’an metropolitan area. They displayed some typical characteristics of grassroots family planning staff across China, i.e., relatively old (averagely aged 44.23 years), less educated (less than half of them obtained a college/university certificate), relatively high mobility (on average, these staff had held the job for less than ten years), and low salary (their salary was around 1500¥ or 220$ a month). About 58% of the staff frequently worked overtime. Most of them had frequent chances of professional training.

Affective organizational commitment seemed to be high, as indicated by all three items. At the same time, their overall job satisfaction and specific satisfactions with working environment, relationship with colleagues, and career prospects were also relatively high; however, the satisfaction with monthly salary was not high. About 34% of the staff members encountered occasional turnover in colleagues; the corresponding figure for frequent turnover was 18%. Most of the staff chose the family planning job, partly because the working place was close to home (75%) or the job was not an obstacle to the care of children (57%). On average, a family planning worker encountered three kinds of difficulties.

The bivariate correlation analysis among the study variables (Appendix B Table A2) supported the hypotheses raised above. For example, specific job satisfactions were associated positively with affective commitment, but negatively with turnover intention. However, to fully test the three sets of hypotheses, the multivariate SEM analysis was needed.

### 3.2. SEM Analysis

Table 2 shows how the predictors mentioned above affected turnover intention. 

First, the affective commitment had a significant negative effect on turnover intention: With each additional unit of organizational commitment, the turnover intention would decrease by 0.261 units (B = −0.261, 95% confidence interval or CI = −0.444, −0.079). The hypothesis H1a was supported. Although the overall job satisfaction also had a negative effect on turnover intention, the effect was not significant (B = −0.097, 95% CI = −0.248, 0.054); thus, the hypothesis H1b was only partly supported. Among the four specific satisfactions, three significantly predicted the affective commitment: Satisfaction with working environment (B = 0.143, 95% CI = 0.057, 0.228); satisfaction with relationship with colleagues (B = 0.360, 95% CI = 0.185, 0.534); satisfaction with career prospects (B = 0.334, 95% CI = 0.234, 0.434). Similarly, three specific satisfactions significantly predicted the overall job satisfaction: Satisfaction with the salary (B = 0.090, 95% CI = 0.025, 0.154); satisfaction with working environment (B = 0.115, 95% CI = 0.044, 0.186); satisfaction with job prospects (B = 0.490, 95% CI = 0.410, 0.571). Thus, the hypothesis H1c was basically supported. Table 3 indicates that specific satisfactions indirectly affected turnover intention, mainly through their effects on affective commitment instead of the overall job satisfaction.

Various individual and organizational factors had either a direct or an indirect effect on turnover intention (Table 2 and Table 3). Age had a significant direct effect on turnover intention: With an increase in age by one year, the turnover intention would decrease by 0.018 units (B = −0.018, 95% CI = −0.035, −0.001). At the same time, its indirect effect via affective commitment was also significant (B = −0.007, 95% CI = −0.012, −0.001). The length of service had a marginally significant negative direct effect (B = −0.017, 95% CI = −0.036, 0.002): For a year increase in service, the turnover intention would decrease by 0.017 units. Frequency of working overtime had a marginally significant direct effect on turnover intention: Compared to the staff never or seldom working overtime, those frequently working overtime had a 0.233 higher intention to quit their current job (B = 0.233, 95% CI = 0.001, 0.464). Opportunity of training had a (marginally) significantly negative indirect effect on turnover intention (frequent vs. none/rare training: B = −0.05, 95% CI = −0.105, 0.005), via its significant positive effect on affective commitment. Thus, the hypotheses H2a and H2b were supported for some individual/organizational factors, especially for age.

Finally, subjective norms about job turnover also had some (direct) effect on turnover intention. Compared to the case where no turnover was seen in colleagues, those staff who encountered frequent turnover in colleagues would have a higher turnover intention (B = 0.278, 95% CI = −0.047, 0.602). Thus, the hypothesis H3a was basically supported. However, no significant effect from any indicator of perceived behavioral control was found. Thus, the hypothesis H3b was not supported.

From the standardized regression coefficients, it can be seen that the important factors that affected turnover intention in family planning staff can be ranked according to the size of the total effect as follows: Age (total effect = direct effect + indirect effect = −0.317), the affective organizational commitment (−0.235), satisfaction with the prospects of family planning services (indirect effect = −0.137), frequency of working overtime (total effect = direct effect + indirect effect = 0.130), frequency of turnover in colleagues (frequent vs. none or seldom: 0.122), length of service (total effect = direct effect + indirect effect = −0.119), satisfaction with relationship with colleagues (indirect effect = −0.054), satisfaction with working environment (indirect effect = −0.052), and opportunity of professional training (total effect = direct effect + indirect effect = −0.049). 

## 4. Discussion

### 4.1. Findings and Discussion

Our study indicates that in a time of higher requirement for family planning services with the introduction of the universal two-child policy in China [2], about 37% of grassroots family planning staff members had either an explicit or an implicit turnover intention in the Xi’an metropolitan area. Furthermore, a series of factors can affect turnover intention in these staff members, which basically supports the hypotheses raised on the basis of our conceptual framework.

First of all, the affective commitment was the central predictor of turnover intention in these family planning workers: On the one hand, it had the largest direct effect; on the other hand, it mediated effects from other factors like age and specific job satisfactions except for satisfaction with pay. By contrast, the overall job satisfaction had no significant effect on turnover intention, and none of the indirect effects via it were significant. Previously, Liu and Mao found that income satisfaction had a significant influence on turnover intention in the grassroots family planning staff in the Beijing city [5]; however, they did not consider affective organizational commitment (note that with our sample, when the affective commitment was excluded from modeling, both the overall job satisfaction and income satisfaction predicted significantly turnover intention; see Table A3 and Table A4 in Appendix C). Evidently, in Maslow’s terms [35], for these family planning workers, satisfaction of higher-level affective needs (esteem, self-actualization, etc.) in work was more important than that of lower-level (cognitive) needs, which partly suggests that the lower-level needs had been satisfied through other channels. For example, their job pay was low and they were not satisfied with it (see Table 1), but there was a vague sign that some of them took family planning services as a part-time job and, thus, received income from other businesses at the same time (this is not allowed, especially in urban areas, and thus, they were reluctant to discuss the details of other incomes; note: Ref. [4] provided some qualitative evidence on the part-time nature of family planning work in rural areas in Shandong, Hubei, and Gansu provinces). More works are warranted to clarify the complicated point.

Second, some individual or organizational factors had an important effect on turnover intention in the family planning staff. Age had a larger total effect than any other factors, and young age was significantly associated with a higher turnover intention, suggesting that the job of family planning services has no attraction for young persons, and thus, there is a challenge to its sustainable development (Table 2 and Table 3). A check of direct vs. indirect effects shows that around one-third of the negative effect of age was through the path of “age→affective commitment→turnover intention.” The positive effect of age on affective commitment seemed to stem partly from a cohort effect. For example, old staff were educated in a time when family planning was emphasized to be highly beneficial to the prosperity of the country, and thus, taking such a job would then bring some sense of esteem and achievement; by contrast, young staff were not cultivated in such an atmosphere, and thus, they may not think family planning is an important public affair. Another important factor that affected turnover intention was length of service: Its indirect effect via affective commitment was positive, although the overall effect was negative (Table 2 and Table 3); in other words, those staff members with longer service were less likely to have an intention to quit their job, not because it was attractive, but for other reasons. The nonattractiveness of the job was also reflected in its requirement of frequent working overtime, which evidently improved turnover intention in these staff members. 

Third, consistent with the theory of planned behavior, our study shows that turnover among colleagues tended to stimulate an intention to quit their job in such family planning staff. We did not measure the injunctive form of subjective norms, e.g., opinions from family members, which might have additional effects on their turnover intention. Contrary to our expectation, the three indicators of perceived behavioral control all had no significant effect on turnover intention. 

Our findings have important implications for the stability and sustainability of China’s family planning services. Simply speaking, the local governments should stabilize the current grassroots family planning staff in the first place by trying to satisfy their basic and higher-level needs (for a preliminary touch upon the topic based on Maslow’s theory, see also ref. [30]); at the same time, the proper friendly packages should be gradually designed to improve the attraction of the job in the long run. More specifically speaking, first, every effort should be made to improve affective commitment among the grassroots staff, given it was the major direct determinant of turnover intention. According to the SEM analysis, as shown in Table 2 and Table 3, such efforts should be invested, first of all, in improving the staff’s confidence in career prospects, including but not limited to, creating a basic medical insurance and retirement package universally for those having worked for more than a given number of years and in improving vocational prestige for them, e.g., formal appointment certificate and special appointment and retirement ceremonies organized by local governments. At the same time, efforts should also be put into improving the working environments and creating a friendly and relaxed working atmosphere. Second, every effort should be made to improve the attraction of family planning services, which is evidently a fundamental challenge, and such efforts should be especially targeted at young people, in the light that age had the largest direct effect on turnover intention. Currently, few persons under 40 and with a higher education experience engage in family planning work in a community/village (Table 1; for a similar observation in other provinces like Gansu, Hebei, Hubei, Jilin, and Shandong, see refs. [4,30,32,33]). This problem has existed for decades and certainly warrants a satisfactory solution. Presumably, the local governments can create some attractive career packages for young staff, e.g., relatively attractive salary and being formally provided a fixed tenure after some years of excellent services. Third, the local governments should make sufficient investment in the professional training of current staff members, in the light that the two-child policy has brought higher requirements for relevant services, which had not been practiced well by such grassroots members before (for an example in Henan province, see ref. [51]). In this aspect, the experience of transformation of family planning work in Hong Kong can be used as an operational reference: An organization will realize a sustainable development only when it continues to satisfy the changing needs of the society [29,52]. Presumably, through the above efforts, turnover frequency can be reduced and mass turnover can be avoided, which is also helpful to attenuate the social multiplier or contagion effect of turnover from colleagues. In the light that maternal and child health is still a challenge to the world, the above suggestions may also shed light on family planning work in other developing countries.

### 4.2. Limitations

There are some limitations with the current study. First, the current study was based on a cross-sectional survey; thus, it was hard to tell how the turnover intention in grassroots staff members had changed with the implementation of the two-child policy. A longitudinal study helps to clarify the point and make the inference of causal relationships more reliable. Second, the sample size of this research was not large, and thus, a finer analysis (e.g., rural vs. urban) was not allowed. Third, the measurements of some predictors of turnover intention can be improved (e.g., subjective norms and quantitative working hours instead of just measuring qualitatively working overtime; see above). Last but not least, the coefficient of determination for turnover intention was not very large, and thus, we may have failed to capture some important predictors. The personality factors may be one of such neglected factors: Previous studies have shown they may have important influence on turnover intention, e.g., by moderating the effects of affective commitment or job satisfaction [53,54]. It is evidently helpful to overcome the above limitations in future studies.

## 5. Conclusions

The current study indicates that in a time of higher requirement for family planning services due to the implementation of the universal two-child policy, nearly one in three grassroots family planning staff had an explicit or implicit turnover intention in the Xi’an metropolitan area, Shaanxi, China. The main factors influencing turnover intention in these staff included (ranked by the size of total effect): Age (direct and indirect effect via affective commitment), the affective commitment (negative effect), specific job satisfactions (i.e., satisfactions with job prospects, relationship with colleagues, and working environment; all had an indirect negative effect via affective commitment), frequency of working overtime (mainly direct positive effect), frequency of turnover among colleagues (direct positive effect), length of service (mainly direct negative effect), and opportunity of professional training (mainly indirect negative effect via affective commitment). Our study suggests that local governments should invest efforts in improving the salary, career prospects, and working environments of grassroots family planning staff members, especially for young staff and in training such staff, to promote sustainable development of the important preventive health-promotion work.

## Figures and Tables

**Table 1:** Descriptive statistics of personal characteristics and other relevant variables.

Group of Variables	Variables/Items	Statistics ^1^
Response variable (1—disagree; 5—strongly agree)	Turnover intention	
	Item 1: Being actively looking for another job	2.00 (0.13)
	Item 2: Being interested in finding a new job this year or next year	1.83 (0.12)
Individual and family characteristics	Place of residence	
	urban	67.54% (5.25%)
	rural	32.46% (5.25%)
	Gender	
	male	1.64% (1.43%)
	female	98.36% (1.43%)
	Age (years)	44.23 (1.22)
	Education	
	pre-college level	53.44% (5.60%)
	college/university or above level	46.56% (5.60%)
Working-related measures	Length of service (years)	9.00 (0.89)
	Monthly salary	2.69(0.14)
	Frequency of working overtime	
	none or seldom	41.97% (5.54%)
	often	58.03% (5.54%)
	Opportunity of training	
	none or seldom	38.69% (5.47%)
	often	61.31% (5.47%)
Affective organizational commitment	Item 1: Feeling a sense of accomplishment	4.21 (0.11)
	Item 2: Being proud to tell others my job	4.24 (0.11)
	Item 3: Feeling respected from doing the work	4.25 (0.10)
Overall job satisfaction	Overall, being satisfied with the current job	4.22 (0.09)
Specific satisfactions	Satisfaction with salary	2.51 (0.12)
	Satisfaction with job environment	4.30 (0.11)
	Satisfaction with job prospects	4.02 (0.10)
	Satisfaction with relationship with colleagues	4.80 (0.05)
Subjective norms	Frequency of colleagues’ turnover	
	never	48.20% (5.61%)
	occasionally	34.10% (5.32%)
	often	17.70% (4.28%)
Perceived behavioral control	Is the working place close to your home?	
	No	25.25% (4.88%)
	Yes	74.75% (4.88%)
	Is it convenient to combine work and care of children?	
	No	42.62% (5.55%)
	Yes	57.38% (5.55%)
	Number of difficulties encountered in work	3.29 (0.16)

^1^ For each categorical variable (e.g., education), ‘Statistics’ refers to the proportion of each category of the variable; for each continuous variable (e.g., age), ‘Statistics’ refers to mean; for each variable, a 95% margin of sampling error is included in brackets.

**Table 2:** The direct effects shown by the SEM modeling.

Group of Predictors	Predictor	Estimate ^1^	95% CI ^2^		Std. All ^3^
			Lower	Upper	
**Response Variable: Turnover Intention (*R*^2^ = 0.244) ^4^**					
Individual and organizational factors	Place of Residence (CG: Rural) ^5^				
	Urban	−0.171	−0.425	0.082	−0.092
	Age (years)	−0.018 *	−0.035	−0.001	−0.220
	Education (CG: Pre-college level)				
	college/university or above level	−0.056	−0.369	0.256	−0.032
	Length of service (years)	−0.017 ^†^	−0.036	0.002	−0.152
	Monthly salary	−0.053	−0.161	0.055	−0.075
	Frequency of working overtime (CG: None or seldom)				
	Often	0.233 *	0.001	0.464	0.132
	Opportunity of training (CG: None or seldom)				
	Often	−0.035	−0.255	0.185	−0.020
Attitude	Affective organizational commitment	−0.261 **	−0.444	−0.079	−0.235
	Overall job satisfaction	−0.097	−0.248	0.054	−0.088
Subjective norms	Frequency of colleagues’ turnover (CG: Never)				
	Occasionally	0.119	−0.130	0.368	0.065
	Often	0.278 ^†^	−0.047	0.602	0.122
Perceived behavioral control	Is the work place close to your home? (CG: No)				
	Yes	0.140	−0.123	0.403	0.070
	Is it convenient to combine work and care of children? (CG: No)				
	Yes	0.062	−0.167	0.291	0.035
	Number of difficulties encountered in work	0.021	−0.052	0.094	0.036
**Response Variable: Affective O** **rganizational Commitment** **(*R*^2^ = 0.503)**					
Individual and organizational factors	Place of residence (CG: Rural)				
	Urban	−0.010	−0.195	0.175	−0.006
	Age (years)	0.025 ***	0.013	0.037	0.351
	Education (CG: Pre-college level)				
	college/university or above level	0.080	−0.137	0.296	0.051
	Length of service (years)	−0.012 ^†^	−0.026	0.002	−0.120
	Frequency of working overtime (CG: None or seldom)				
	Often	0.046	−0.123	0.215	0.029
	Opportunity of training (CG: None or seldom)				
	Often	0.191 *	0.029	0.353	0.119
Specific satisfactions	Satisfaction with salary	0.046	−0.032	0.124	0.064
	Satisfaction with job environment	0.143 **	0.057	0.228	0.170
	Satisfaction with job prospects	0.334 ***	0.234	0.434	0.378
	Satisfaction with relationship with colleagues	0.360 ***	0.185	0.534	0.207
**Response Variable: Overall Job Satisfaction (*R*^2^ = 0.526)**					
Individual and organizational factors	Place of residence (CG: Rural)				
	Urban	−0.137 ^†^	−0.290	0.015	−0.081
	Age (years)	0.012 *	0.003	0.022	0.171
	Education (CG: Pre-college level)				
	college/university or above level	0.065	−0.114	0.244	0.041
	Length of service (years)	−0.006	−0.017	0.006	−0.059
	Frequency of working overtime (CG: None or seldom)				
	Often	−0.090	−0.229	0.050	−0.056
	Opportunity of training (CG: None or seldom)				
	Often	0.016	−0.118	0.150	0.010
Specific satisfactions	Satisfaction with salary	0.090 **	0.025	0.154	0.123
	Satisfaction with job environment	0.115 **	0.044	0.186	0.137
	Satisfaction with job prospects	0.490 ***	0.410	0.571	0.550
	Satisfaction with relationship with colleagues	0.094	−0.049	0.237	0.054

^1 †^*p* < 0.1; * *p* < 0.05; ** *p* < 0.01; *** *p* < 0.001; ^2^ CI—95% confidence interval; ^3^ std. all—standardized regression coefficient; ^4^ *R*^2^—the coefficient of determination; ^5^ CG, comparison group.

**Table 3:** The indirect effects shown by the SEM modeling.

Group of Predictors	Path ^1^	Estimate ^2^	95% Cl ^3^		Std. All ^4^
			Lower	Upper	
Individual and organizational factors	TI←AOC←Place of residence	0.003	−0.046	0.051	0.001
	TI←AOC←Age	−0.007 *	−0.012	−0.001	−0.082
	TI←AOC←Education	−0.021	−0.079	0.038	−0.012
	TI←AOC←Length of service	0.003	−0.001	0.007	0.028
	TI←AOC←Frequency of working overtime	−0.012	−0.057	0.033	−0.007
	TI←AOC←Opportunity of training	−0.050 ^†^	−0.105	0.005	−0.028
Specific satisfactions	TI←AOC←Satisfaction with salary	−0.012	−0.034	0.010	−0.015
	TI←AOC←Satisfaction with job environment	−0.037 *	−0.071	−0.003	−0.040
	TI←AOC←Satisfaction with job prospects	−0.087 **	−0.153	−0.022	−0.089
	TI←AOC←Satisfaction with relationship with colleagues	−0.094 *	−0.173	−0.015	−0.049
Individual and organizational factors	TI ← OJS ←Place of residence	0.013	−0.012	0.039	0.007
	TI ← OJS ←Age	−0.001	−0.003	0.001	−0.015
	TI ← OJS ←Education	−0.006	−0.026	0.014	−0.004
	TI←OJS←Length of service	0.001	−0.001	0.002	0.005
	TI←OJS←Frequency of working overtime	0.009	−0.010	0.028	0.005
	TI←OJS←Opportunity of training	−0.002	−0.015	0.012	−0.001
Specific satisfactions	TI←OJS←Satisfaction with salary	−0.009	−0.024	0.006	−0.011
	TI←OJS←Satisfaction with job environment	−0.011	−0.030	0.007	−0.012
	TI←OJS←Satisfaction with job prospects	−0.048	−0.122	0.027	−0.048
	TI←OJS←Satisfaction with relationship with colleagues	−0.009	−0.029	0.011	−0.005

^1^ TI refers to turnover intention, AOC refers to affective organizational commitment, and OJS refers to the overall job satisfaction; ^2 †^ *p* < 0.1; * *p* < 0.05; ** *p* < 0.01; ^3^ CI—95% confidence interval; ^4^ std. all—standardized regression coefficient.

## Appendix A

**Table A1:** The coding of predictors.

Predictors	Description
Individual characteristics	
place of residence	0—rural; 1—urban
personal age	Actual age of the respondent
education level	0—pre-college level; 1—college/university level or above
Work-related factors	
length of service	Actual service years of the respondent
monthly salary	1—less than 1000¥; 2—1000~1500¥; 3—1500~2000¥; 4—2000~2500¥; 5—2500~3000¥; 6—3000~3500; 7—more than 3500¥ ^1^
frequency of working overtime	0—none or seldom; 1—often
opportunity of training	0—none or seldom; 1—often
Affective organizational commitment	1—disagree; 2—somewhat disagree; 3—neither agree nor disagree; 4—relatively agree; 5—strongly agree
Overall job satisfaction	1—not satisfied; 2—somewhat not satisfied; 3—neutral; 4—relatively satisfied; 5—highly satisfied
Specific job satisfactions	1—disagree; 2—somewhat disagree; 3—neither agree nor disagree; 4—relatively agree; 5—strongly agree
Subjective norm	1—none; 2—occasionally; 3—often
Factors limiting or promoting quitting the current job	
Proximity of working place to home	0—no; 1—yes
Convenience of combining work and childcare	0—no; 1—yes
Practical difficulties in working (six in total)	0—no; 1—yes for each difficulty

^1^ Note: The exchange rate of RMB to dollar at the time of survey was about 1:7.

## Appendix B

**Table A2:** The correlation among the study variables.

	**1 ^1^**	**2**	**3**	**4**	**5**	**6**	**7**	**8**	**9**	**10**	**11**	**12**	**13**	**14**	**15**	**16**	**17**
1	—																
2	0.032	—															
3	−0.354 ***	−0.276 ***	—														
4	0.200 **	0.436 ***	−0.647 ***	—													
5	−0.302 ***	−0.315 ***	0.709 ***	−0.413 ***	—												
6	0.153 *	0.408 ***	−0.473 ***	0.601 ***	−0.334 ***	—											
7	−0.028	−0.306 ***	0.372 ***	−0.365 ***	0.346 ***	−0.201 **	—										
8	−0.136 *	−0.091	0.246 ***	−0.203 **	0.295 ***	−0.165 **	0.211 ***	—									
9	−0.360 ***	−0.209 **	0.380 ***	−0.343 ***	0.243 ***	−0.446 ***	0.198 **	0.252 ***	—								
10	−0.256 ***	−0.254 ***	0.247 ***	−0.289 ***	0.162 **	−0.351 ***	0.082	0.116 *	0.591 ***	—							
11	−0.150 *	−0.279 ***	0.208 ***	−0.293 ***	0.189 **	−0.186 **	0.159 **	0.030	0.341 ***	0.408 ***	—						
12	−0.087	−0.096 ^†^	0.059	−0.119 *	0.075	−0.158 **	0.062	0.071	0.371 ***	0.362 ***	0.297 ***	—					
13	−0.209 **	−0.225 ***	0.188 **	−0.307 ***	0.126 *	−0.308 ***	0.091	0.136 *	0.569 ***	0.681 ***	0.379 ***	0.311 ***	—				
14	−0.128 ^†^	−0.131 *	0.058	−0.117 *	0.080	−0.141 *	0.086	0.101 ^†^	0.379 ***	0.267 ***	0.181 **	0.223 ***	0.279 ***	—			
15	0.225 **	0.287 ***	−0.327 ***	0.413 ***	−0.225 ***	0.388 ***	−0.212 ***	−0.054	−0.327 ***	−0.303 ***	−0.339 ***	−0.193 **	−0.327 ***	−0.109 ^†^	—		
16	0.014	−0.048	0.080	−0.048	0.027	−0.222 ***	0.026	0.034	0.208 **	0.150 *	0.179 **	0.086	0.105 ^†^	−0.002	−0.195 **	—	
17	0.106	−0.045	−0.169 **	0.047	−0.114 *	−0.059	−0.075	0.064	0.021	0.119 *	0.042	0.102 ^†^	0.098 ^†^	0.109 ^†^	0.021	0.415 ***	—
18	0.150 *	0.054	−0.100 ^†^	0.173 **	−0.022	0.073	0.025	0.058	−0.229 ***	−0.178 **	−0.341 ***	−0.164 **	−0.203 ***	−0.153 **	0.311 ***	−0.016	0.073

^1^ † *p* < 0.1; * *p* < 0.05; ** *p* < 0.01; *** *p* < 0.001. 1—turnover intention; 2—place of residence; 3—age; 4—education; 5—length of service; 6—monthly salary; 7—frequency of working overtime; 8—opportunity of training; 9—affective organizational commitment; 10—overall job satisfaction; 11—satisfaction with salary; 12—satisfaction with working environment; 13—satisfaction with job prospects; 14—satisfaction with relationship with colleagues; 15—frequency of colleagues’ turnover; 16—the proximity of work place to home; 17—the convenience in combining work and care of children; 18—number of difficulties encountered in work.

## Appendix C. SEM Analysis without Considering the Affective Commitment

**Table A3:** The direct effects shown by the SEM modeling (the affective commitment was not considered).

Group of Predictors	Predictive	Estimate ^1^	95% CI ^2^		Std. All ^3^
			Lower	Upper	
**Response Variable: Turnover Intention (*R*^2^ = 0.210) ^4^**					
Individual and organizational factors	Place of residence (CG: rural) ^5^				
	Urban	−0.173	−0.422	0.077	−0.095
	Age (years)	−0.021 *	−0.038	−0.005	−0.270
	Education (CG: pre-college level)				
	college/university or above level	−0.080	−0.386	0.227	−0.046
	Length of service (years)	−0.014	−0.033	0.004	−0.130
	Monthly salary	−0.024	−0.130	0.082	−0.034
	Frequency of working overtime (CG: none or seldom)				
	Often	0.192 ^†^	−0.035	0.418	0.111
	Opportunity of training (CG: none or seldom)				
	Often	−0.090	−0.302	0.123	−0.051
Attitude	Overall job satisfaction	−0.201 **	−0.342	−0.061	−0.186
Subjective norms	Frequency of colleagues’ turnover (CG: never)				
	Occasionally	0.107	−0.138	0.351	0.059
	Often	0.294 ^†^	−0.026	0.614	0.131
Perceived behavioral control	Is the work place close to your home? (CG: no)				
	Yes	0.106	−0.152	0.363	0.054
	Is it convenient to combine work and care of children? (CG: no)				
	Yes	0.067	−0.157	0.292	0.039
	Number of difficulties encountered in work	0.037	−0.034	0.109	0.064
**Response variable: Overall Job Satisfaction (*R*^2^ = 0.526)**					
Individual and organizational factors	Place of residence (CG: rural)				
	Urban	−0.137 ^†^	−0.290	0.015	−0.081
	Age (years)	0.012 *	0.003	0.022	0.171
	Education (CG: pre-college level)				
	college/university or above level	0.065	−0.114	0.244	0.041
	Length of service (years)	−0.006	−0.017	0.006	−0.059
	Frequency of working overtime (CG: none or seldom)				
	Often	−0.090	−0.229	0.050	−0.056
	Training opportunity (CG: none or seldom)				
	Often	0.016	−0.118	0.150	0.010
Specific satisfactions	Satisfaction with salary	0.090 **	0.025	0.154	0.123
	Satisfaction with job environment	0.115 **	0.044	0.186	0.137
	Satisfaction with job prospects	0.490 ***	0.410	0.571	0.550
	Satisfaction with relationship with colleagues	0.094	−0.049	0.237	0.054

^1 †^*p* < 0.1; * *p* < 0.05; ** *p* < 0.01; *** *p* < 0.001; ^2^ CI—95% confidence interval; ^3^ std. all—standardized regression coefficient; ^4^ *R*^2^—the coefficient of determination; ^5^ CG, comparison group.

**Table A4:** The indirect effects shown by the SEM modeling (the affective commitment was not considered).

Group of Predictors	Path ^1^	Estimate ^2^	95% Cl ^3^		Std. All ^4^
			Lower	Upper	
Individual and organizational factors	TI←OJS←Place of residence	0.028	−0.009	0.064	0.015
	TI←OJS←Age	−0.003 ^†^	−0.005	0.000	−0.032
	TI←OJS←Education	−0.013	−0.050	0.024	−0.008
	TI←OJS←Length of service	0.001	−0.001	0.004	0.011
	TI←OJS←Frequency of working overtime	0.018	−0.013	0.049	0.010
	TI←OJS←Opportunity of training	−0.003	−0.030	0.024	−0.002
Specific satisfactions	TI←OJS←Satisfaction with salary	−0.018 ^†^	−0.036	0.000	−0.023
	TI←OJS←Satisfaction with job environment	−0.023 *	−0.045	−0.002	−0.025
	TI←OJS←Satisfaction with job prospects	−0.099 **	−0.170	−0.028	−0.102
	TI←OJS←Satisfaction with relationship with colleagues	−0.019	−0.051	0.013	−0.010

^1^ TI refers to turnover intention, and OJS refers to the overall job satisfaction; ^2 †^ *p* < 0.1; * *p* < 0.05; ** *p* < 0.01; ^3^ CI—95% confidence interval; ^4^ std. all—standardized regression coefficient.
